# A complete chloroplast genome of *Rubia yunnanensis* Diels (Rubiaceae), a traditional Chinese herb endemic to China

**DOI:** 10.1080/23802359.2022.2107454

**Published:** 2022-08-10

**Authors:** Shuying Zhao, Haiying Liang, Peng Tang, John K. Muchuku

**Affiliations:** a College of Environment and Ecology, Jiangsu Open University (The City Vocational College of Jiangsu), Nanjing, People’s Republic of China; b Department of Botany, Jomo Kenyatta University of Agriculture and Technology, Nairobi, Kenya

**Keywords:** Rubiaceae, *Rubia yunnanensis*, chloroplast genome, phylogenetics

## Abstract

*Rubia yunnanensis* Diels 1912 (Rubiaceae) is a plant used in traditional Chinese medicine. We here assembled a complete chloroplast (cp) genome for *R. yunnanensis* using Illumina HiSeq reads. The genome is 155,108 bp in length. The genome contains 113 genes, including 79 protein coding genes, 30 tRNA genes, and four rRNA genes. The large single-copy (LSC) region is 84,848 bp, inverted repeat A (IRa) region is 26,573 bp, small single-copy (SSC) region is 17,114 bp, and inverted repeat B (IRb) region is 26,573 bp. A phylogenomic analysis found that *R. yunnanensis* is close to *R. cordifolia*. The assembled cp genome in this study provided a basis for the conservation and phylogenetic studies of *R. yunnanensis*.

*Rubia yunnanensis* Diels (Rubiaceae) is endemic to Yunnan and Sichuan provinces, China; herbs, perennial; stem usually clumped, sometimes procumbent, 10–50 cm (Flora of China; http://www.efloras.org/flora_page.aspx?flora_id=2); rootstock and somewhat thickened storage roots, red, famous as ‘xiaohongshen’. Roots of *R. yunnanensis* are used as medicines and natural dyes in Yunnan province.

This article is licensed under laboratory work regulations of Jiangsu Open University, Nanjing city, Jiangsu province, China. *Rubia yunnanensis* was collected from Kunming (25.159 N; 102.76 E; alt. 2,100 m), Yunnan, China in July 2020. The voucher was deposited at the Herbarium of China Pharmaceutical University (voucher number LYC-2007, Lingyun Chen, lychen83@qq.com). Genomic DNA was extracted from leaves using a modified procedure of CTAB (cetyltrimethyl ammonium bromide). A library of Illumina sequencing with an average fragment length of 350 bp was constructed and then sequenced using the BGISEQ-500 platform (150 × 2 bp) at the Novogene Co. Ltd. (Tianjin, China). Adapters and low-quality bases were removed using Trimmomatic v0.27 (SLIDINGWINDOW:4:15 LEADING:5 TRAILING:4 MINLEN:80; Bolger et al. 2014). The chloroplast (cp) genome was assembled using GetOrganelle v1.7.5 (Jin et al. 2020) with the parameter ‘embplant_pt’ and default parameters. The genome was annotated using the software PGA (Qu et al. 2019) with annotations of *Amborella trichopoda* (AJ506156), *Helianthus annuus* (NC_007977), and *Nerium oleander* (NC_025656) as references and default parameters.

The whole length is 155,108 bp. Annotation found 113 genes, including 79 protein coding genes, 30 tRNA genes, and four rRNA genes. The large single-copy (LSC) region is 84,848 bp, inverted repeat B (IRa) region is 26,573 bp, small single-copy (SSC) is 17,114 bp, and inverted repeat B (IRb) region is 26,573 bp. The overall GC content is 36.98%. The GC content of the LSC, IRa, SSC, and IRb regions is 34.50%, 42.87%, 30.96%, and 42.87%, respectively.

To appraise the phylogenetic position of *R. yunnanensis*, cp genomes of 21 species belonging to Spermacoceae alliance of Rubiaceae were accessed. Chloroplast genomes were aligned using MAFFT v7.407 (Katoh and Standley 2013) and low occupancy columns were trimmed using Phyutility v.2.7.1 (-clean 0.01). Then, a maximum-likelihood (ML) inference with the concatenated matrix was carried out using RAxML v8.2.12 (Stamatakis 2006) with the GTRCAT model and 200 rapid bootstrap replicates. The analysis found that *R. yunnanensis* is sister to *R. cordifolia* with bootstrap value (BS)=100 (Figure 1). Rubia formed a clade with *Galium* with BS = 100. These results are consistent with previous studies, such as Yang et al. (2018). The cp genome of *R. yunnanensis* provided a useful resource for the conservation of this species and phylogenetic research of Rubiaceae.

**Figure 1.**
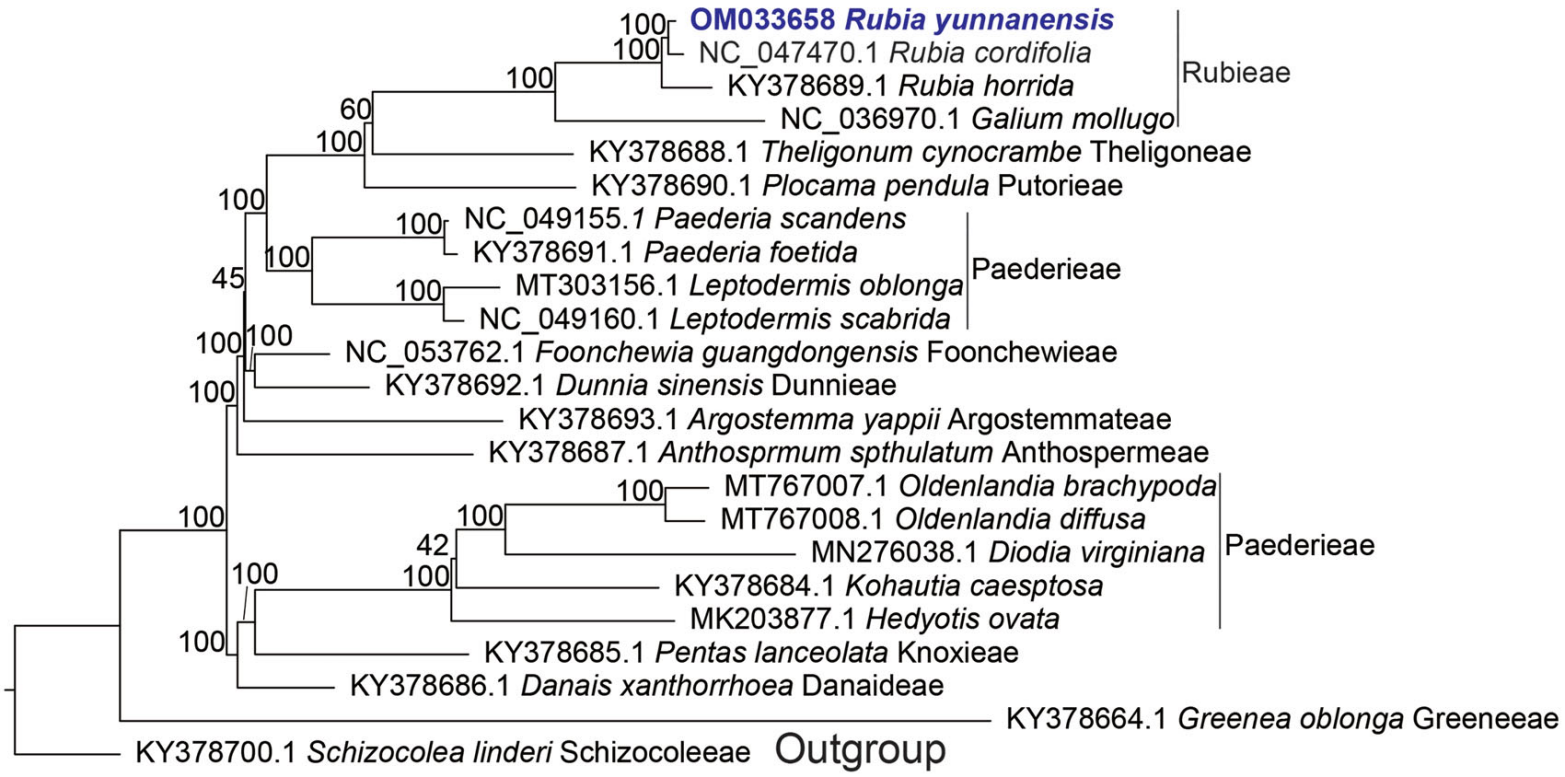
A maximum-likelihood phylogeny of 23 Rubiaceae species, constructed using the CDSs of 81 chloroplast genes (*accD*, *atpA*, *atpB*, *atpE*, *atpF*, *atpH*, *atpI*, *ccsA*, *cemA*, *clpP*, *infA*, *matK*, *ndhA*, *ndhB*, *ndhC*, *ndhD*, *ndhE*, *ndhF*, *ndhG*, *ndhH*, *ndhI*, *ndhJ*, *ndhK*, *petA*, *petB*, *petD*, *petG*, *petL*, *petN*, *psaA*, *psaB*, *psaC*, *psaI*, *psaJ*, *psbA*, *psbB*, *psbC*, *psbD*, *psbE*, *psbF*, *psbH*, *psbI*, *psbJ*, *psbK*, *psbL*, *psbM*, *psbN*, *psbT*, *psbZ*, *rbcL*, *rpl14*, *rpl16*, *rpl20*, *rpl2*, *rpl22*, *rpl23*, *rpl32*, *rpl33*, *rpl36*, *rpoA*, *rpoB*, *rpoC1*, *rpoC2*, *rps11*, *rps12*, *rps14*, *rps15*, *rps16*, *rps18*, *rps19*, *rps2*, *rps3*, *rps4*, *rps7*, *rps8*, *ycf1*, *ycf15*, *ycf2*, *ycf3*, *ycf4*, *ycf68*). Each species name follows its tribal classification and GenBank number.

## Data Availability

The data that support the findings of this study are openly available in GenBank of NCBI at https://www.ncbi.nlm.nih.gov under the accession no. OM033658. The associated BioProject, SRA, and Bio-Sample numbers are PRJNA805178, SRS11974251, and SAMN25829797, respectively.
